# Clinical analysis of Primary Gastrointestinal Non-Hodgkin’s Lymphoma

**DOI:** 10.12669/pjms.336.13631

**Published:** 2017

**Authors:** Wei Wang, Peng Lin, Haiying Yao, Xi Jia, Jirui Sun

**Affiliations:** 1 Wei Wang, Baoding First Central Hospital, Baoding 071000, Hebei Province, China; 2 Peng Lin, Baoding First Central Hospital, Baoding 071000, Hebei Province, China; 3 Haiying Yao, Baoding First Central Hospital, Baoding 071000, Hebei Province, China; 4 Xi Jia, Baoding First Central Hospital, Baoding 071000, Hebei Province, China; 5 Jirui Sun, Baoding First Central Hospital, Baoding 071000, Hebei Province, China

**Keywords:** Gastrointestinal tract, non-Hodgkin’s lymphoma, Prognosis

## Abstract

**Objective:**

To study the clinical characteristics and prognostic factors of survival for patients with primary gastrointestinal non-Hodgkin’s lymphoma (PGI-NHL).

**Methods:**

A retrospective analysis was performed for 104 PGI-NHL patients who were admitted in Baoding First Central Hospital from July 2003 to January 2014.

**Results:**

There were 58 males and 46 females. The median age of onset was 53 (15-83) years old. In terms of pathogenic sites, there were 51 gastric cases (49.00%) and 53 intestinal cases (51.00%), with the median survival of 35 (1-130) months. The 1-, 3- and 5-year overall survival (OS) rates were 88.40%, 80.70% and 78.80%, respectively. The factors influencing the progression-free survival (PFS) and OS rates were studied by univariate and multivariate analyses. The PFS and OS rates of patients with B-cell PGI-NHL were significantly higher than those of patients with T-cell PGI-NHL (P<0.05). The PFS and OS rates of patients with primary gastric lymphoma were significantly higher than those of patients with primary intestinal lymphoma (P<0.05), but the data were not associated with Ann Arbor stage or LDH level (P>0.05). Compared with non-surgical patients, only patients with intestinal lymphoma benefited from surgery (P<0.05).

**Conclusion:**

The pathogenic site and pathological type are risk factors that affect the survival of PGI-NHL patients, and chemotherapy should be given the first priority for patients with primary gastric lymphoma.

## INTRODUCTION

Primary gastrointestinal non-Hodgkin lymphoma (PGI-NHL) is a common malignancy of the gastrointestinal tract, which belongs to extranodal lymphoma. The lesion can occur in any part of the digestive tract from the mouth to the anus, of which stomach and ileocecal junction are the most common pathogenic sites, accounting for 30%-45% of all extranodal lymphomas.^1^-^3^ Digestive tract is the most common involved part of extranodal lymphomas. Digestive tract lymphoma can be either primary in the gastrointestinal tract, or secondary in other parts of lymphoma invasions. In this study, we retrospectively analyzed the clinical data of 104 patients with PGI-NHL treated in Baoding First Central Hospital from July 2003 to January 2014. Its incidence, clinical manifestations and the effects of treatment on progression-free survival (PFS) and overall survival (OS) were analyzed.

## METHODS

This study was approved by the ethics committee of Baoding First Central Hospital, and written consent was obtained from all patients. A total of 104 patients with PGI-NHL diagnosed by pathologists in Baoding First Central Hospital from July 2003 to January 2014 were enrolled in this study. Among them, 51 cases occurred in the stomach and 53 cases in the intestinal tract. The data of examinations were collected from all the patients during confirmation, including blood routine, blood biochemistry (including LDH), electrocardiogram, chest and abdomen CT or PET/CT, bone marrow cell morphology, cardiac ultrasound, gastroscopy and colonoscopy. Pathological types were classified according to the 2001 WHO Blood Lymphoma Diagnostic Criteria, and identified by the pathologists in Baoding First Central Hospital through histopathological and immunohistochemical examinations. Clinical staging was conducted according to the Ann Arbor staging criteria and the Lugano staging system of gastrointestinal lymphoma. The follow-up deadline was May 1st, 2014. PFS refers to the period from the beginning of treatment to the observed progression of the disease or the occurrence of death for any reason. OS refers to the time from diagnosis to the last follow-up or death.

### Treatment regimens

Of the 104 patients, 45 (43.27%) received surgery and chemotherapy, 50 (48.08%) single chemotherapy, and 8 (7.69%) combined metronidazole, clarithromycin and omeprazole therapy against *Helicobacter pylori* (HP) [8 cases of mucosa-associated lymphoid tissue borderline area B cell lymphoma (MALT lymphoma)], and one case (0.96%) local radiotherapy. The chemotherapy regimen was systemic chemotherapy (6 to 8 cycles) mainly with rituximab (R) combined with CHOP (cyclophosphamide plus Adriamycin plus vincristine plus prednisone) regimen or CHOP regimen.

Of the 51 primary gastric patients, 13 (25.49%) received surgery plus chemotherapy, 30 (58.82%) single chemotherapy, and 8 (15.69%) anti-HP treatment. Of the 53 primary intestinal patients, 32 (60.38%) received surgery plus chemotherapy, 20 (37.73%) single chemotherapy, and 1 (1.89%) local radiotherapy.

Of the 104 patients, 54 (51.92%) were treated with rituximab, 42 did not receive glucocorticoids in the process of the CHOP regimen for the first time, and 32 were given a reduced dose of glucocorticoid.

### Statistical analysis

All data were analyzed by SPSS 18.0 software. Prognostic factors of survival were subjected to univariate analysis with the Kaplan-Meier method. Inter-group comparisons were conducted by the Log-rank test. Multivariate analysis was carried out by the Cox regression model. P<0.05 was considered statistically significant.

## RESULTS

### Clinical characteristics

The 104 patients included 58 males and 46 females, with a median onset age of 53 (15 to 83) years old. There were 33 cases (31.73%) of gastrointestinal bleeding, seven cases (6.73%) of organ perforation, and six cases of obstruction (5.77%). Among the 104 patients, 101 cases received morphological examination of bone marrow cells and biopsy histopathological examination, of which 5 cases (4.95%) appeared bone marrow invasions. Forty-nine patients underwent HP infection examination (including lesion biopsy, serum HP antibody, 13C breath test, etc.), of which 26 cases (53.06%) were positive. In terms of three to five points (43.27%). Among the 104 patients, 29 (27.88%) were at Ann Arbor stage I/II and 75 (72.12%) at stage III/IV. In the 51 patients with primary gastric disease, 13 cases (25.49%) were in Lugano stage IE, 14 cases (27.45%) in stage IIE and 24 cases (47.06%) in stage IV. Among the 53 patients with primary intestinal disease, there were 4 cases (7.55%) in stage I E, 10 cases (18.87%) in stage II E, and 39 cases (73.58%) in stage IV. LDH levels were normal in 79 cases (75.96%), and increased in 25 cases (24.04%).

### Pathological types

In the 104 patients, there were 18 cases (17.31%) of T cell phenotype, and 86 cases of B cell phenotype (82.69%). Among them, there were 53 cases (50.96%, 14 deaths) of diffuse large B cell lymphoma (DLBCL), 26 cases of MALT lymphoma (25.00%, one death), 4 cases (3.85%) of mantle cell lymphoma, one case (0.96%) of follicular lymphoma (FL), two cases (1.92%, one death for acute myocardial infarction) of small lymphocyte lymphoma, 6 cases (5.77%, two deaths) of peripheral T cell lymphoma, one case (0.96%) of anaplastic large cell lymphoma, eight cases (7.69%, 6 deaths) of enteropathy-associated T-cell lymphoma, two cases (1.92%, one death) of angioimmunoblastic T-cell lymphoma, and one case (0.96%, one death) of nasal extranodal NK/T cell lymphoma.

### Survival analysis

Until May 1, 2014, the median of follow-up was 35 (1 to 130) months. Of the 104 patients, 26 (25.00%) died, 10 of whom were primary NHL and 16 primary intestinal NHL. Of the 26 patients, 16 died of disease progression, seven died of myelosuppressive infection after chemotherapy, 1 died of second tumor (liver cancer), one died of acute myocardial infarction, and four cases (8.00%) appeared severe gastrointestinal complications of gastrointestinal bleeding and perforation. Twenty-six patients (57.78%) underwent postoperative complications such as various degrees of early satiety, delayed gastric emptying, alkaline reflux gastritis, dumping syndrome, anastomotic stenosis and incomplete intestinal obstruction. The 1-year, 3-year and 5-year OS rates of all patients were 88.40%, 80.70% and 78.80%, respectively; those of 51 primary gastrointestinal patients were 90.20%, 82.40% and 80.40%, respectively; and those of primary intestinal patients were 90.60%, 79.20% and 69.80%, respectively.

**Fig.1:**
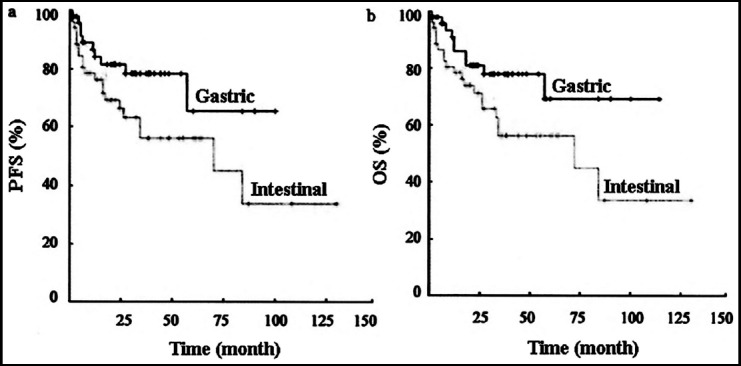
PFS (a) and OS (b) of PGI-NHL patients at different pathogenic sites.

The patients were divided into the stomach group and the intestine group according to the differences in the pathogenic sites. The age, gender, Ann Arbor stage, LDH level, IPI score, cell phenotype, and Lugano stage were compared between the two groups. Statistically significant differences were founded in LDH level, Ann Arbor stage, cell phenotype and Lugano stage (P<0.01) (Table-I). The two groups were analyzed by the Kaplan-Meier method, and the results showed that the differences of PFS and OS rates between the two groups were statistically significant (P=0.043 and 0.047, respectively) (Fig.1).

**Table-I:** Clinical characteristics of PGI-NHL patients at different pathogenic sites.

*Clinical characteristic*	*Case No.*	*Stomach*	*Intestinal tract*	*χ^2^*	*P*
Gender				0.930	0.335
Male	58	26 (44.8)	32 (55.2)		
Female	46	25 (54.3)	21 (45.7)		
Age (year)				0.315	0.575
<60	62	29 (46.8)	33 (53.2)		
≥60	42	22 (52.4)	20 (47.6)		
Ann Arbor stage				8.792	0.003
I~II	29	21 (72.4)	8 (27.6)		
III~IV	75	30 (40.0)	45 (60.0)		
LDH level (IU/L)				14.740	0.000
<270	79	41 (51.9)	38 (48.1)		
≥270	25	10 (40)	15 (60.0)		
IPI score				1.475	0.225
0~2 points	59	32 (54.2)	27 (45.8)		
3~5 points	45	19 (42.2)	26 (57.8)		
Cell phenotype				9.128	0.004
B cell	86	48 (55.8)	38 (44.2)		
T cell	18	3 (16.7)	15 (83.3)		
Lugano stage				7.658	0.006
I~II	41	27 (65.9)	14 (34.1)		
III~IV	63	24 (38.1)	39 (61.9)		

Multivariate analysis was performed for cell phenotype, Ann Arbor stage, LDH, Lugano stage, primary site, IPI score and treatment regimen. Only cell phenotype was the influencing factor for PFS and OS (P<0.05) (Table-II).

**Table-II:** Multivariate analysis for survival factors of PGI-NHL patients.

*Factor*	*PFS*			*OS*		

	*HR*	*95%CI*	*P*	*HR*	*95%CI*	*P*
Cell phenotype	5.281	1.457~19.145	0.011	5.531	1.230~17.682	0.004
Ann Arbor stage	1.760	0.000~5.880	0.947	2.340	0.000~2.360	0.953
Lugano stage	1.474	0.457~4.750	0.516	1.075	0.375~3.085	0.893
LDH	2.832	1.144~7.008	0.024	2.582	0.992~6.724	0.052
Single use of rituximab	0.457	0.165~1.261	0.130	0.624	0.228~1.704	0.357
Pathogenic site	0.703	0.297~7.008	0.423	0.580	0.242~1.390	0.222

All the 104 patients were divided into the T cell group and the B cell group according to the cell phenotype. There was no significant difference between the two groups (P = 0.301). The Kaplan-Meier method was used to analyze the survival of the two groups. The results showed that the difference in the PFS and OS rates were statistically significant between the two groups (P=0.000), suggesting that the survival of patients with B-cell lymphoma was significantly better than that of T-cell lymphoma patients.

Further stratification analysis showed that the PFS and OS rates of the DLBCL patients treated with rituximab were significantly better than those who did not receive rituximab administration. (P = 0.007 and 0.039, respectively). The 51 primary gastric patients were divided into the surgery group, the non-surgery group and the single anti-HP group, and the results showed that the PFS and OS differences were not statistically significant in the three groups (P = 0.192 and 0.174, respectively). The 53 primary intestinal patients were divided into the surgery plus chemotherapy group and the single chemotherapy group, and the results showed that the PFS and OS rates of the surgery plus chemotherapy group were significantly better than those of the single chemotherapy group, between which the difference was statistically significant (P = 0.035 and 0.009, respectively) (Table-III).

**Table-III:** Factors affecting PFS and OS of PGI-NHL patients (x ± s).

*Group*	*Case No.*	*PFS*		*OS*	

		*Time (month)*	*P*	*Time (month)*	*P*
Pathological phenotype			0.000		0.000
T cell group	18	27.7±7.7		31.0±7.4	
B cell group	86	98.5±7.7		96.7±7.4	
Single use of rituximab for DLBCL patients			0.007		0.039
Without use group	14	52.6±14.2		32.2±7.2	
Use group	39	98.9±9.8		95.7±10.2	
Primary gastric patients			0.192		0.174
Surgery group	30	32.4±10.3		32.4±10.3	
Non-surgery group	13	25.3±3.8		25.3±3.8	
Anti-HP group	8	35.2±3.4		35.2±3.4	
Primary intestinal patients			0.035		0.009
Surgery plus chemotherapy group	32	79.8±12.1		84.7±10.7	
Chemotherapy group	21	41.3±7.7		33.4±6.9	

## DISCUSSION

PGI-NHL is a group of malignant lymphoid hematopoietic tissue tumors primarily occurring in lymphoid follicles in the gastrointestinal tract, which is mostly found in stomach compared with intestinal tract. The pathogenic site of stomach, small intestine and rectum and diffuse lesion of colon accounts for 50% -70%, about 35%, and 4%-6%, respectively, of which the gastrointestinal tract can be involved at the same time.^4^,^5^

PGI-NHL occurs frequently at the age of 50 to 60 years old, with the clinical manifestations similar to other gastrointestinal tumors, the majority of which is B-cell lymphoma. According to foreign literature statistics, B-cell phenotype accounts for more than 90% of gastrointestinal lymphomas.^6^ Song et al.^7^ summarized the data of 101 PGI-NHL patients, and found that B and T lymphocyte phenotypes accounted for 90.09% and 9.91%, respectively. In this study, we found that T and B lymphocyte phenotypes accounted for 17.31% and 82.69%, respectively, slightly lower than the results in the domestic and foreign literature. DLBCL and MALT lymphomas are the most common pathological patterns of PGI-NHL,^8^ and FL is very rare in PGI-NHL, with an incidence of less than 7%.^9^ Studies have confirmed that gastric MALT lymphoma is closely related to HP infection.^10^ The NCCN treatment guidelines suggest that HP-positive I/II gastric MALT lymphoma patients should receive anti-HP antibiotic therapy as initial treatment, but must conduct strict serology and endoscopic follow-up. For HP-negative or advanced patients, the treatment principle is the same as that for FL patients. Due to the application of anti-CD20 antibody rituximab, DLBCL patients have experienced a higher survival rate in recent years. Although the role of HP infection in patients with gastric MALT lymphoma has been recognized, its impact on gastric DLBCL patient’s remains unsure.^11^-^13^ The primary incidence of males in the intestinal tract was significantly higher than that in females, with a ratio of 2.5:1. HP infection, high fat and high protein diet, and environmental pollution are all considered to be associated with the incidence of intestinal patients.

We conducted the single factor analysis on 104 patients with PGI-NHL, and the results showed that there were significant differences in Ann Arbor stage, Lugano stage, LDH level and cell phenotype between intestinal and gastric origins. Song et al.^7^ found that the prognosis of patients with intestinal origin was significantly worse than those with gastric origin. Our findings were the same. Regardless of single factor analysis or multivariate analysis, the survival status of T cell phenotype was significantly worse than that of B cell phenotype.

So far, we have not found a factor that can clearly assess the prognosis of patients with PGI-NHL. Previous reports have indicated the factors that may affect prognosis, such as age, gender, symptoms, LDH levels, and IPI scores, but their values are still controversial, IPI scores in particular.^14^-^16^

In the past, the resection of total or most gastrectomy is the therapeutic method that is used the most commonly. Its 5-year survival rate of patients may reach more than 80%. It has been possible to retain the stomach function due to the effect of anti-HP treatment and the application of rituximab in recent years, and the role of surgery can be re-evaluated. Our study showed that only 8% of non-surgical patients suffered from severe gastrointestinal complications after chemotherapy and received emergency surgery, and 57.78% of patients appeared various degrees of gastrointestinal complications after surgery, indicating that chemotherapy has obvious advantages in terms of safety and long-term quality of life. And in the early stages of onset, although 31.73% of patients had symptoms of gastrointestinal bleeding such as hematemesis and melena, chemotherapy did not increase the risk of gastrointestinal bleeding. Currently, the role of surgical treatment in primary bowel patients remains controversial. Khosla et al.^17^ retrospectively analyzed 27 patients with primary intestinal lymphoma, and found that the 5-year OS rate of patients receiving surgery and chemotherapy reached 79.5%, significantly better than 13.9%, that of patients undergoing single chemotherapy. The primary gastric patients are not recommended for surgery, and advanced patients who appear gastrointestinal bleeding, obstruction or perforation and other complications generally choose surgical treatment.

### Authors’ contributions

**WW & PL:** Designed this study and wrote this paper.

**HY, XJ & JS:** Performed this study and analyzed results.
